# PRF and PRP in Dentistry: An Umbrella Review

**DOI:** 10.3390/jcm14093224

**Published:** 2025-05-06

**Authors:** Alfonso Acerra, Mario Caggiano, Andrea Chiacchio, Bruno Scognamiglio, Francesco D’Ambrosio

**Affiliations:** Department of Medicine, Surgery and Dentistry, University of Salerno, 84084 Salerno, Italy; macaggiano@unisa.it (M.C.); a.chiacchio6@studenti.unisa.it (A.C.);

**Keywords:** autologous platelet concentrates, dentistry, oral surgery, platelet-rich fibrin, platelet-rich plasma

## Abstract

**Introduction**: Platelet-rich fibrin (PRF) and platelet-rich plasma (PRP) utilize autologous blood and share the objective of leveraging blood-derived growth factors to enhance the body’s natural healing process. A large extensive use has been made in various branches of dentistry. **Methods**: A total of 4175 records were identified from the electronic search, specifically 291 from BioMed Central, 3406 from MEDLINE/PubMed, 304 from the Cochrane library databases, and 174 from the PROSPERO register. This review was performed in relation to the PRISMA flow chart and was annotated in the PROSPERO register. **Results**: In total, 3416 title abstracts were screened, and a total of 40 systematic reviews were finally included in the present umbrella review. **Conclusions**: Research supports the use of PRF and PRP in different fields of dentistry. This is a huge potential for the patient but also for the doctor as these products are from the patient and have zero cost. However, further studies are needed, especially RCTs, to have clearer evidence on the role of PRF and PRP.

## 1. Introduction

The first generation of platelet concentrates is represented by platelet-rich plasma (PRP), first documented in the late 1990s [1]. Although a wide variety of protocols for PRP preparation have been proposed, they generally all require two main steps: centrifugation and activation. Once collected in a tube with anticoagulant, the blood undergoes a first centrifugation to separate the plasma from the red blood cells (RBCs), and then, the plasma undergoes a second centrifugation to separate the platelets from the plasma (PRP fraction = platelet-rich plasma and PPP fraction = platelet-poor plasma). The platelet pellet along with some leukocytes are suspended in a smaller volume of PPP and activated by thrombin, calcium chloride (CaCl_2_), or type I collagen (e.g., from the soft tissue at the patient’s surgical site). Through this double-centrifugation process, platelets are enriched approximately 2–10 times compared to normal blood [2]. Over the past years, numerous attempts have been made to standardize the PRP preparation/protocol, with significant variation among studies regarding spin speed, centrifugation time, blood volume, anticoagulants, and coagulation activators; so, it is difficult to directly compare the reported results [3]. Platelet-rich fibrin (PRF) is a second-generation of platelet concentrates following platelet-rich plasma (PRP) [4]. Both PRF and PRP utilize autologous blood and share the objective of leveraging blood-derived growth factors to enhance the body’s natural healing process. PRF builds upon PRP by preserving growth factors within a fibrin matrix, allowing for it to exert its effects over several days or weeks post-surgery. Unlike PRP, PRF is created without the use of anticoagulants, which are known to impede wound healing. Compared to PRP, PRF preparations generally have a higher concentration of leukocytes due to advances in centrifugation techniques, have a fibrin matrix that facilitates healing while enabling a gradual release of growth factors, and are available in different forms to enhance usability. The leukocyte-PRF (L-PRF) is obtained by single centrifugation of blood collected in a tube without anticoagulant or activators. This protocol makes preparation simpler, less expensive, and less risky by obtaining a material with a high concentration of leukocytes, which contribute to immune and antibacterial responses [5]. In particular, the original L-PRF protocol involves a single centrifugation cycle (10 min at 3000 RPM). Solid PRF was the original version developed by Choukroun and colleagues [6]. To achieve solid forms (clots/membranes), the use of glass blood tubes is mandatory. The combination of spontaneous coagulation and centrifugation leads to the formation of a fibrin clot in which platelets and leukocytes are trapped. The final L-PRF product, after gentle compression of the clot, is a rather strong fibrin matrix (L-PRF membrane) with a concentration of white blood cells and platelets more than 20 times higher (compared to their concentration in the patient’s original blood). These membranes offer several advantages:

- Release of numerous growth factors important for tissue regeneration/healing, lasting up to 14 days;
- Promotion of angiogenesis;
- Provision of antibacterial properties;
- Increased graft stability when mixed with a bone substitute;
- Support for soft tissue healing.

There is evidence of differences in gender and timing of preparation of L-PRF [6]. Miron et al. studied the incidence of gender on macroscopic characteristics of L-PRF membranes and observed that females produced membranes 17% larger than males [7]. Larger membranes were also detected in older patients. It is also a well-known fact that a short timing between harvesting and centrifugation is mandatory to have a high-quality membrane. In particular, the cut-off is preferably <1 min. If the time between blood sampling and centrifugation increases to 3 min, the clots become significantly smaller, and after 5 min, a small incoherent, friable mass of fibrin is formed instead [8].

A sufficient blood supply is essential for wound healing and is one of the key factors that can affect the overall outcome, whether it leads to regeneration or repair. Hemostasis and the formation of a fibrin clot are the initial steps that activate the wound healing process. Platelets are the first cells to arrive at the wound site and play a role in the hemostatic phase of healing. Once they bind to von Willebrand factor and collagen in the subendothelial matrix, platelets become activated. While their main function is hemostasis, platelets also contribute to inflammation by releasing proteolytic enzymes and cationic proteins from their granules [3].

The release of these activated platelets leads to the discharge of over 300 bioactive molecules, including various growth factors such as platelet-derived growth factor (PDGF), vascular endothelial growth factor (VEGF), insuline-like growth factor (IGF-2), epidermal growth factor (EGF), and tumor growth factor-β (TGF-β). These molecules help attract immune cells and osteogenic precursors, acting as messengers and regulators that influence a wide range of interactions between cells and the extracellular matrix. As a result, autologous blood proteins (like growth factors) can support the formation of new blood vessels (angiogenesis) as well as the growth and maturation of tissues. Throughout the wound healing process, platelets stick to the injured area, triggering platelet-to-platelet interactions (aggregation) under the regulation of plasma mediators, such as epinephrine, thrombin, and substances released by activated platelets (adenosine diphosphate (ADP) and serotonin). Both platelets and leukocytes are essential players in the body’s innate and adaptive immune responses. Therefore, the key biological rationale for using an autologous blood product, such as a platelet concentrate, is to concentrate and deliver growth factors, cytokines, lysosomes, and cells derived from the blood into the wound environment. This ultimately enhances the healing process in both soft and hard tissues, promoting the body’s natural capacity to heal.

There are different types of PRF, each with unique characteristics and clinical applications. The primary types include L-PRF, a form of PRF that retains leukocytes and is created without anticoagulants. It is primarily used in solid form for wound healing and tissue regeneration. Its strength lies in its gradual release of growth factors over time. Then, there is injectable-PRF (i-PRF), a liquid form of PRF that stays injectable for a limited period (15–20 min). Compared to PRP, it provides a slower and more prolonged release of growth factors, enhancing healing [9]. Concentrated-PRF (C-PRF) instead concentrates platelets and leukocytes more effectively through higher-speed centrifugation. It allows for a significant increase in the concentration of cells and growth factors, which makes it highly effective for healing and regeneration. The primary differences between these types lie in their form (solid vs. liquid), duration of action, and concentration of growth factors and cells, each tailored for specific medical applications

The main difference between PRF types lies in the centrifugation protocols used and the use of hydrophobic tubes, which significantly affect the concentration of cells and growth factors. L-PRF typically uses low-speed centrifugation (about 2700 rpm for 12 min), which retains a good number of leukocytes and platelets. This protocol helps form a solid clot, which gradually releases growth factors over a longer period.

i-PRF utilizes a shorter, slower centrifugation (700 rpm for 3–4 min). This allows for the PRF to remain in a liquid state for about 15–20 min, suitable for procedures requiring injectability, such as mixing with bone grafts. The slower centrifugation preserves more growth factors but has a limited working time before clotting [10].

C-PRF involves high-speed centrifugation (2000 RCF for 8 min) to maximize the concentration of platelets and leukocytes in the PRF. This technique ensures a denser, more potent layer of PRF, concentrating cells in the buffy coat for higher regenerative potential.

These differences in centrifugation protocols (speed, duration, and rotor type) determine the cell concentration and clot formation, optimizing PRF for specific clinical applications.

Although data in the literature are scarce and controversial, the purpose of this systematic literature review is to investigate the most used prp and prf in dentistry, evaluating the effectiveness of the use of APCs in the various therapies described [10].

## 2. Materials and Methods

### 2.1. Study Protocol

The study protocol was developed in accordance with the PRISMA (Preferred Reporting Items for Systematic Reviews and Meta-analyses) flow chart [11] before the literature search, data extraction, and analysis and was registered on the PROSPERO systematic review register (ID: 1023323, 31 March 2025), as recommended by Booth et al. [12].

The research question was focused on the following:

Population: human subjects who have undergone oral surgery with PRF or PRF;

Exposure: effect of PRP and PRF;

Outcomes: evaluation of the effect of PRF and PRP in oral surgery, oral regenerative, Onj, periodontology, and implantology.

### 2.2. Search Strategy

Systematic reviews (with or without meta-analyses) published in the English language concerning how prf or prp influence oral surgery were electronically searched until 1 August 2024 across the PROSPERO register and Scopus, MEDLINE/PubMed, BioMed Central, and the Cochrane Library databases by two independent reviewers (A.A. and A.C.), combining the following keywords with Boolean operators:

(‘PRF’ OR ‘PRP’ OR ’Platelet Rich Plasma’ OR ‘Platelet Rich Fibrin’) AND (‘Oral Surgery’ OR ‘Implantology’ OR ‘Dentistry’).

The following filters were applied: “Review (English)” on the Scopus database; “Systematic Review (English)” on the MEDLINE/PubMed database; “Keywords” on the Cochrane library. No filters were employed on the BioMed Central database, Scopus database, and PROSPERO register.

Data collection was conducted in the main scientific search engines, including articles from the last 10 years, to obtain results only for newer medications.

### 2.3. Eligibility Criteria

The results were screened according to the defined eligibility criteria; inclusion and exclusion criteria were defined during the study design, as shown in the Table 1.

### 2.4. Study Resarch

Collected citations were recorded, duplicates were eliminated through the Zootero reference manager tool, and the remaining titles were screened by two independent reviewers (A.A. and A.C.). The same two reviewers subsequently screened relevant abstracts of systematic reviews with or without meta-analyses.

The full texts of those potentially eligible title abstracts were obtained, and the full texts were independently reviewed by the same authors (A.A. and A.C.). Any disagreement was solved by a discussion, and a third author (B.S.) was consulted in case of doubts.

The reference lists of the included reviews were also screened for relevant titles, and the subsequent study screening was performed as already described.

No restrictions regarding the date of publication, number of studies, and study design were included in each systematic review, and the number of dental implants and kind of restorations were applied.

### 2.5. Data Extraction and Collection

Data were independently extracted on a standardized data extraction form by two reviewers (A.A. and A.C.), who reached consensus by discussion, also involving a third author (B.S.) when needed.

From each of the systematic reviews with or without meta-analyses included in the present umbrella study, the following data meeting the eligibility criteria were recorded, when available:

- First author, year, journal, funding, and quality of the study;
- Number and design of the studies included in each systematic review;
- Characteristics of oral intervention with PRF or PRP;
- Outcomes;
- Conclusions.

### 2.6. Data Synthesis

A narrative synthesis of the data concerning the investigated population, exposure, and outcomes was conducted. Data from the included studies were qualitatively synthesized through descriptive statistical analysis using Microsoft Excel 2019 (Microsoft Corporation, Redmond, WA, USA).

### 2.7. Quality Assessment

The quality assessment of the systematic reviews presently included was performed with the Assessing the Methodological Quality of Systematic Reviews (AMSTAR) 2 tool, accessed online (https://amstar.ca) 19 August 2022, evaluating for quality the systematic reviews of randomized and/or nonrandomized studies [13].

## 3. Results

### 3.1. Study Selection

A total of 4175 records were identified from the electronic search, specifically 291 from BioMed Central, 3406 from MEDLINE/PubMed, 304 from the Cochrane library databases, and 174 from the PROSPERO register.

In total, duplicates were eliminated, and 3416 title abstracts were screened.

Of these 3416 title abstracts, only 98 abstracts were relevant for the present systematic review; so, the full texts were screened, and 58 articles were further excluded, specifically because they (*n* = 6) were not relevant (*n* = 52) or did not meet the inclusion criteria, as shown in Table 2.

**Figure 1:**
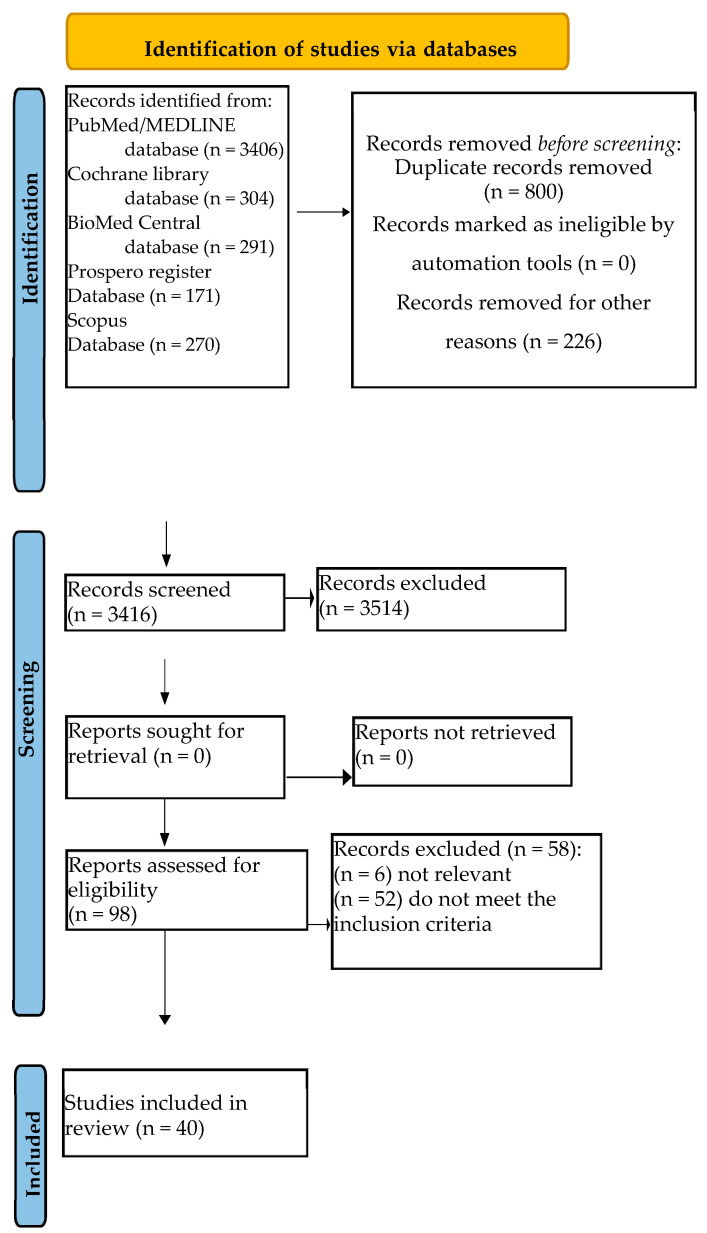
The Prisma flow chart.

A total of 40 systematic reviews were finally included in the present umbrella review (Figure 1).

The characteristics and evaluation from included studies are summarized in Table 3, Table 4, Table 5, Table 6, Table 7, Table 8, Table 9 and Table 10.

### 3.2. Quality and Risk of Bias Assessment of Included Systematic Review

Many studies were classified as low or moderate quality, and one was classified as critically low quality, using the Assessing the Methodological Quality of Systematic Reviews (AMSTAR) 2 tool, as illustrated in Table 11.

## 4. Discussion

Forty systematic reviews were included in this study, evaluating the use of platelet-rich plasma (PRP) and platelet-rich fibrin (PRF) in different branches of dentistry. This use, recently widely described in the literature, seems to have important results on the quality and rate of tissue healing, especially soft tissue, even improving postoperative symptoms such as pain and inflammation.

### 4.1. Oral Surgery

The extraction of the third molar is one of the most prevalent surgical procedures in oral surgery. It is also associated with a high incidence of postoperative sequelae, all related to the inflammatory state that arises following the procedure. The primary postoperative symptomatic manifestations include pain, swelling, difficulty in mastication, and prolonged healing time of the site. Ten reviews were identified regarding the use of PRP and PRF in the extraction of impacted lower third molars.

All included studies agree that PRF reduces postoperative complications, significantly alleviates pain and swelling, and decreases the incidence of alveolar osteitis following the extraction of an impacted lower third molar. However, no significant differences were observed between the PRF and non-PRF groups concerning osteoblastic activity and, consequently, bone healing. Furthermore, most studies concur on the reduction in postoperative trismus incidence and better soft tissue healing, inflammation reduction, and acceleration of wound healing. In contrast, the study by Xiang et al. did not demonstrate any difference in soft tissue healing when using PRF compared to its absence; it also showed no effect on reducing postoperative trismus [80].

Regarding postoperative bleeding following dental extraction in patients undergoing anticoagulant therapy, the study by Campana et al. indicated that anticoagulated patients receiving PRF without the discontinuation of their medication experienced reduced postoperative bleeding and shorter hemostasis times [82]. This finding is further corroborated by the study by Filho et al. In addition, patients exhibited a reduction in pain and accelerated wound healing [71].

Oral-antral communication (OAC) is a complication that can occur when a tooth, especially upper molars, is extracted, resulting in the alveolus extending to create an opening between the oral cavity and the maxillary sinus. This can lead to issues such as infections, sinusitis, and healing difficulties. Salgado-Peralvo et al. demonstrated that the use of PRF alone for the closure of OAC resulted in a 100% success rate when the diameter of the OAC was up to 5 mm [75]. This finding is significant, as PRF provides a solution to a type of complication that typically requires more invasive surgical techniques for repair and treatments to manage potential infections or complications.

### 4.2. Implantology

The stability of the implant is considered a primary factor in achieving clinical success with osseointegrated implants. Only two reviews identified the use of PRF in relation to the stability of osseointegrated implants. According to these studies, the application of PRF and its subtype L-PRF appears to promote the formation of new bone around the implant. This effect may be attributed to the regenerative properties of the growth factors present in PRF, which stimulate the healing process and neoangiogenesis. Additionally, it seems to enhance the secondary stability of the implant by reducing the loading time, potentially leading to improved clinical outcomes and greater patient satisfaction.

### 4.3. Periodontology

Periodontal disease causes a loss of supporting hard and soft tissues of the tooth; so, periodontal regeneration aims to repair and rebuild these tissues. Among the most regenerable defects are intrabony defects, which represent a loss of vertical alveolar bone tissue adjacent to the root of the teeth. Starting with non-surgical therapy, Niemczyk et al. described the effectiveness of i-PRF in combination with scaling and root planning in improving clinical parameters, with an interesting role of i-PRF as bactericidal action against Porphyromonas Gingivalis [111].

Castro et al. observed a significant reduction in probing depth (PD) and an increase in clinical attachment level (CAL) and bone fill when comparing L-PRF with open flap debridement. Additionally, a review by Miron et al., which included 27 randomized controlled trials (RCTs), highlighted that the combination of open flap debridement (OFD) and platelet-rich fibrin (PRF) yielded better results in terms of CAL and PD than OFD alone. Furthermore, no differences were reported among the groups OFD/barrier membrane [BM], OFD/PRP, and OFD/enamel matrix derivatives [EMD] when compared to OFD/PRF. No improvements were noted when PRF was added to OFD/EMD.

One of the greatest challenges in periodontology is the therapy of furcations, those areas between the roots of multi-rooted teeth that, because of their anatomy, are difficult to clean in the first steps of therapy and difficult to regenerate in subsequent steps. In particular, complex management occurs because furcation defects are lesions surrounded by non-vascularized structures, the roots of the teeth, and thus unable to provide the blood supply for regeneration, resulting in an unpredictable procedure.

Castro et al. and Skurska et al. agree that the use of platelet-rich fibrin (PRF) in conjunction with conventional open flap debridement (OFD) has demonstrated statistically significant advantages in terms of probing depth (PD), clinical attachment level (CAL), and bone fill [89,90].

The use of platelet-rich fibrin (PRF) in combination with the coronally advanced flap (CAF) surgical technique may represent, as described by Miron et al., an effective treatment modality for gingival recessions that exhibit adequate width of keratinized mucosa (KMW) at the base [87]. This is supported by data indicating that the use of PRF in combination with CAF significantly improves the percentage of root coverage compared to CAF alone; however, it does not enhance KMW.

As highlighted by Panda et al., the data regarding its efficacy in covering gingival recessions do not show statistically significant results [92]. This suggests that, while PRF may offer some benefits, it is not universally effective for all cases of gingival recession. In contrast, Castro et al. demonstrated that PRF compared to connective tissue grafting (CTG) did not show significant differences in terms of PD reduction, CAL gain, recession reduction, and keratinized tissue width (KTW) [90]. Moreover, reviews concerning its application at the palatal graft site indicate significant advantages. According to Gusman et al. and Meza-Mauricio et al., the use of PRF in this context was associated with reduced postoperative pain, potentially translating to lower analgesic consumption by patients [88,91]. Furthermore, PRF appears to promote greater and more rapid wound healing, facilitating early re-epithelialization at the donor site.

These factors render PRF a promising option in the management of soft tissues in periodontology, particularly in surgical procedures where pain and healing are critical considerations.

### 4.4. ONJ

Osteonecrosis of the jaws is a severe complication associated with various medications, not only bisphosphonates but also other agents such as vascular endothelial growth factor (VEGF) inhibitors and anti-resorptive drugs. This has led to an increased need for a better understanding of the management of this condition, especially in patients requiring surgical interventions, such as dental implant placement or extractions.

The use of autologous platelet concentrates, such as platelet-rich fibrin (PRF) and platelet-rich plasma (PRP), has been investigated as a treatment option as well as a preventive measure in at-risk patients. There are currently no definitive guidelines governing their use, and the evidence regarding their efficacy is still being gathered and evaluated. The approach may differ depending on whether the focus is prevention to mitigate the risk of osteonecrosis in patients on certain medications or therapy for treating cases that are already in an advanced stage of the condition.

Only one article met the inclusion criteria for this systematic review. According to Rusilas et al., the use of PRP or PRF leads to faster wound closure (after one month) and a reduction in the risk of infection at the surgical site [93]. Additionally, there was a lower need for re-intervention in the PRF group as well as reduced pain and discomfort. These findings are corroborated by the study conducted by Ghanaati et al., who additionally demonstrated better healing outcomes when PRF was combined with bone morphogenetic protein 2 (BMP2) [112]. A great innovation for these patients could be the use of PRF as a carrier for antibiotics. A review by Niemczyk et al. showed that this could be an aid and a solution that should be more investigated with further research [113].

### 4.5. Regenerative

Bone resorption following tooth extraction is a complex process that involves changes in bone cells and growth factors. The absence of the tooth root, which normally stimulates the bone remodeling process, leads to a loss of bone volume, particularly in the initial months post-extraction.

The implications of this bone resorption can be significant. Firstly, the reduction in bone volume can complicate the placement of dental implants, as adequate bone tissue is essential to support the implant [114]. Additionally, resorption may affect facial aesthetics, resulting in a loss of facial contour in the affected area. Various surgical techniques have been developed to mitigate and reduce bone resorption [115,116].

Studies on the use of platelet-rich plasma (PRP) and platelet-rich fibrin (PRF) to decrease post-extraction alveolar bone resorption are controversial. According to Moraschini et al., the application of platelet concentrates appears to accelerate healing and epithelialization of soft tissues in extraction sites and reduce postoperative pain and discomfort [98]. Moreover, it enhances the gain of keratinized gingiva following soft tissue surgery and decreases pain and inflammation, thus providing greater postoperative comfort; however, it has not demonstrated effects on bone regeneration. This data have also been corroborated by Lin et al., who indicated that PRF alone in ridge preservation does not provide significant additional benefits compared to natural healing cavities regarding bone volume, bone density, and osteoblastic activity [105].

Conversely, other studies, such as reviews by Pan et al. and Dragonas et al., suggest that PRF may be associated with less variation in mesial bone height, reduced bone resorption, and greater bone fill following tooth extraction [102,104]. Furthermore, the review by Al-Maawi et al. indicates that, in 85% of cases, the filling of the oral cavity was superior in the PRF group compared to spontaneous wound healing [97]. According to Niu et al., PRF might have a more favorable impact on the preservation of alveolar width and height compared to PRP [94].

Anitua et al. observed reduced horizontal and vertical bone loss during the early stages of healing, particularly in the 2–3 months following extraction, when applying PRP or PRF at the tooth extraction site [103]. However, it was noted that an extended healing period may not confer additional benefits.

Moreover, according to Caponio et al., the use of L-PRF and P-PRP in alveolar ridge preservation is advantageous, as any platelet concentrate enhances new bone formation compared to spontaneous healing [106]. Del Fabbro et al. demonstrated that alveoli filled with PRF or PRP exhibited superior quality new bone formation and significantly higher mineral density compared to natural clots [100].

### 4.6. Endodontics

The use of APC (autologous platelet concentrate) in the field of endodontics has represented a noteworthy advancement in the management of apical lesions and associated complications. These cellular therapies, due to their regenerative and anti-inflammatory properties, offer significant potential in promoting the healing of periapical tissues and in the regeneration of bone tissues [117].

The applications of APC in endo/periosteal lesions, endodontic surgery, and apical procedures have shown promising results, as evidenced by numerous clinical studies.

According to the study by Meschi et al., APC in endodontic treatments appears to contribute to the healing of soft and hard tissues, improve patients’ quality of life in the early postoperative period, facilitate further root development, and support the maintenance or recovery of pulp vitality [107]. However, the lack of standardized criteria for assessing the quality of healing represents a significant limitation. Further research is needed to validate these findings.

### 4.7. Orthodontics

Only one article was identified in this field. According to the meta-analysis by Farshidfar et al., inconsistent results among the various APCs are highlighted: PRP shows no significant effects, i-PRF presents positive results, while L-PRF offers conflicting results [108]. i-PRF seems to accelerate dental movement, particularly in the second month, but does not show significant effects in the third month. Differences in preparation methods, administration, and the quantity of APCs may influence the results. Despite the potential benefits, the variability of results and the risk of bias require further well-designed studies to confirm these effects, as also evidenced by a review on surgical procedures in accelerating orthodontic movements [118].

### 4.8. Oral Lesion

Only two articles were identified in this field, both focusing on the use of PRF (platelet-rich fibrin) and PRP (platelet-rich plasma) in the treatment of oral lichen planus (OLP). Oral lichen planus (OLP) is a chronic inflammatory condition of the oral mucosa that presents various therapeutic challenges. The range of manifestations, from reticular lesions to ulcerations, necessitates careful clinical evaluation and a treatment plan tailored to the specific needs of the patient. A multidisciplinary approach, often involving dermatologists, dentists, and other specialists, can be beneficial for optimizing symptom management and improving the patient’s quality of life [119]. In addition to corticosteroids, which remain the standard treatment due to their effectiveness in reducing inflammation and pain, there are other pharmacological agents and therapeutic strategies that may be considered.

Maddheshiya et al. demonstrated that the use of PRP had a significant efficacy in alleviating the clinical signs and symptoms associated with oral lichen planus that appeared resistant to conventional treatment, without adverse reactions [109]. Furthermore, the results of the review by Gupta et al. suggest that i-PRF (injectable platelet-rich fibrin) is promising for reducing pain levels and the sensation of burning, improving lesion size, and increasing patient satisfaction among those affected by OLP [110].

## 5. Conclusions

Research supports the use of PRF and PRP in different fields of dentistry. There are several branches in which the use of APCs is recognized to give efficacy in terms of clinical outcomes and postoperative symptoms, so that its use is part of daily clinical practice, such as in oral surgery. However, there are other procedures in which the use of APCs needs further investigation and clinical studies clarifying the real benefit and branches in which its use has still been poorly investigated. Furthermore, the heterogeneity of the included studies, such as the lack of parameters that were used to obtain APCs, and the risk of bias should be considered. Despite the limitations and an increased requirement for clinical trials in some therapy or procedures, the use of APCs demonstrates a huge potential for the patient’s healing and also for the doctor, as these products are from the patient and at zero cost without any risk.

## Figures and Tables

**Table 1:** Inclusion and exclusion criteria.

Inclusion Criteria	Exclusion Criteria
Systematic review about only human subjects who have undergone oral surgery or a dental procedure with only autologous platelet concentrates (PRF * or PRP *)	Not a systematic review with or without meta-analysis
Systematic review with or without meta-analysis	Not an English language article
Only English articles	Not an in vitro or animal systematic review
Only reviews published in the last ten years	Does not meet the inclusion criteria
	Not relevant (missing data)
	Reviews published more than ten years ago

* PRF = platelet-rich fibrin; PRP = platelet-rich plasma.

**Table 2:** Studies excluded and reasons.

Reference	Reasons for Exclusion
[14]	did not meet the inclusion criteria
[15]	did not meet the inclusion criteria
[16]	did not meet the inclusion criteria
[17]	did not meet the inclusion criteria
[18]	not relevant (missing data)
[19]	did not meet the inclusion criteria
[20]	did not meet the inclusion criteria
[21]	did not meet the inclusion criteria
[22]	did not meet the inclusion criteria
[23]	did not meet the inclusion criteria
[24]	did not meet the inclusion criteria
[25]	did not meet the inclusion criteria
[26]	did not meet the inclusion criteria
[27]	did not meet the inclusion criteria
[28]	did not meet the inclusion criteria
[29]	did not meet the inclusion criteria
[30]	did not meet the inclusion criteria
[31]	did not meet the inclusion criteria
[32]	did not meet the inclusion criteria
[33]	did not meet the inclusion criteria
[34]	did not meet the inclusion criteria
[35]	did not meet the inclusion criteria
[36]	did not meet the inclusion criteria
[37]	did not meet the inclusion criteria
[38]	did not meet the inclusion criteria
[39]	did not meet the inclusion criteria
[40]	did not meet the inclusion criteria
[41]	did not meet the inclusion criteria
[42]	did not meet the inclusion criteria
[43]	did not meet the inclusion criteria
[44]	did not meet the inclusion criteria
[45]	did not meet the inclusion criteria
[46]	did not meet the inclusion criteria
[47]	did not meet the inclusion criteria
[48]	did not meet the inclusion criteria
[49]	did not meet the inclusion criteria
[50]	did not meet the inclusion criteria
[51]	did not meet the inclusion criteria
[52]	not relevant (missing data)
[53]	did not meet the inclusion criteria
[54]	did not meet the inclusion criteria
[55]	not relevant
[56]	not relevant
[57]	did not meet the inclusion criteria
[58]	did not meet the inclusion criteria
[59]	did not meet the inclusion criteria
[60]	did not meet the inclusion criteria
[61]	not relevant
[62]	did not meet the inclusion criteria
[63]	did not meet the inclusion criteria
[64]	did not meet the inclusion criteria
[65]	did not meet the inclusion criteria
[66]	did not meet the inclusion criteria
[67]	did not meet the inclusion criteria
[68]	not relevant
[69]	did not meet the inclusion criteria
[70]	did not meet the inclusion criteria

**Table 3:** The characteristics and evaluation from included studies in oral surgery.

Authors, Year Reference Journal Study Design	PRF or PRP and Oral Surgery Application	Evaluation	Conclusions
Filho et al., 2021 [71] J. of Oral and Maxillofacial surgery Systematic review and meta-analysis	PRF and PRP after tooth extraction in patients using oral anticoagulant therapy	Bleeding after extraction Pain Alveolitis after extraction	The use of PRF did not improve the risk of bleeding, pain score, and alveolitis after extraction.
Bao et al., 2021 [72] West China Journal of Stomatology Systematic review and meta-analysis	PRF after mandibular third molar extraction	Pain Swelling Soft tissue healing Trismus Alveolar osteitis Bone healing	PRF effectively reduced pain after extraction, attenuated post-extraction swelling, and promoted soft tissue healing. PRF significantly reduced trismus and alveolar osteitis. No positive effect was reported on bone healing vs. control group.
Franchini et al., 2019 [73] Blood transfusion Systematic review and meta-analysis	PRP in periodontal bone defects	PD CAL Gingival recession Bony defect	PRP was slightly more effective compared to control groups in all the outcomes described.
Al-Hamed et al., 2017 [74] J. of Oral and Maxillofacial surgery Systematic review and meta-analysis	PRF after mandibular third molar extraction	Pain Trismus Swelling PD Soft tissue healing Incidence of localized osteitis Bone healing	Positive results were recorded for all the outcomes described, but no beneficial role was reported in bone healing.
Salgado-Peralvo et al., 2022 [75] J Stomatol Oral Maxillofac Surg Systematic review	PRF in oroantral communication	Pain Soft tissue healing Number of analgesic OAC closure	PRF group had significantly lower pain and number of analgesics than control group. There was OAC closure in 100% of cases using PRF alone when diameter was up to 5 mm.
He et al., 2017 [76] J. of Oral and Maxillofacial Surgery Systematic review and meta-analysis	PRF during lower third molar extraction	Wound healing Osseous and soft tissue regeneration Pain Swelling Alveolar osteitis	Local application of PRF after lower third molar extraction is a valid method for relieving pain and 3-day postoperative swelling and reducing the incidence of alveolar osteitis.
Vitenson et al., 2022 [77] J. of Oral Maxillofacial Surgery Systematic review and meta-analysis	A-PRF after mandibular third molar extraction	Pain Facial swelling Trismus Soft tissue healing	A-PRF resulted in lower pain scores after 2, 3, and 7 days, had a negligible effect on facial swelling and trismus, and seems to have some beneficial effect on soft tissue healing.
Bao et al., 2021 [78] J. of Oral Maxillofacial Surgery Systematic review and meta-analysis	L-PRF and A-PRF in mandibular third molar extraction	Postoperative pain Soft tissue healing	Application of A-PRF after third molar extraction had the best effect in improving postoperative pain after 3 and 7 days, and L-PRF promoted the degree of soft tissue healing after 7 days.
Ramos et al., 2022 [79] J. of Oral and Maxillofacial surgery Systematic review and meta-analysis	PRF, A-PRF, and L-PRF in impacted lower third molar extraction	Pain Edema Trismus Soft tissue healing Periodontal regeneration adjacent to the second molar	The use of L-PRF and A-PRF allows for better control of pain and edema, but neither has an effect on trisumus. PRF and L-PRF protocols improve soft tissue healing and probing depth at the third month after third molar surgery.
Xiang et al., 2019 [80] BMC Oral Health Systematic review and meta-analysis	PRF in mandibular third molar surgery	Pain Swelling Trismus Osteoblastic activity Soft tissue healing Alveolar osteitis	PRF only reduces some of the postoperative complications; it significantly relieved the pain and swelling and reduced the incidence of alveolar osteitis after the extraction of an impacted lower third molar, but no significant differences were revealed between PRF and non-PRF groups in trismus, osteoblastic activity and soft tissue healing.
Canellas et al., 2017 [81] Int J. of Oral and Maxillofacial Surgery Systematic review and meta-analysis	PRF in mandibular third molar surgery	Postoperative pain Alveolar osteitis Swelling Bone healing	PRF reduced postoperative pain and swelling and showed a decrease in prevalence of alveolar osteitis. Further investigation needs to be conducted to assess the real effect of the PRF on bone healing.
Campana et al., 2023 [82] Journal of clinical medicine Systematic review	APC (L-PRF, A-PRF) after tooth extraction in patients on anticoagulant therapy	Postoperative bleeding Pain Wound healing	Patients on anticoagulant therapy who received APCs without discontinuing their medication experienced decreased postoperative bleeding, a shorter hemostasis time, reduced pain, and accelerated wound healing.
Zhu et al., 2021 [83] Int J. of Oral and Maxillofacial Surgery Systematic review and meta-analysis	PRF in mandibular third molar surgery	Alveolar osteitis Pain Trismus Soft tissue healing Swelling	The use of PRF reduced the incidence of alveolar osteitis and postoperative pain following third molar surgery. Furthermore, PRF may also improve postoperative soft tissue healing.
Riberio et al., 2024 [84] Clin Oral Investig. Systematic review	L-PRF in third molar surgery	Pain Edema Postoperative healing	L-PRF plays an important role in reducing postoperative pain, edema, and the incidence of alveolar osteitis and infections.

APC = autologous platelet concentrates; PRF = platelet-rich fibrin; PRP = platelet-rich plasma; PD = probing depth; CAL = clinical attachment loss; OAC = oroantral communication; A-PRF = advanced platelet-rich fibrin; L-PRF = leucocyte platelet-rich fibrin.

**Table 4:** The characteristics and evaluation from included studies in implantology.

Authors, Year Reference Journal Study Design	Application	Evaluation	Conclusions
Lyris et al., 2021 [85] J Stomatol Oral Maxillofac Surg Systematic review and meta-analysis	* L-PRF in implant bed prior to implant placement	Implant stability quotient (ISQ) *	L-PRF had a positive effect on secondary implant stability.
Guan et al., 2023 [86] Heliyon Systematic review and meta-analysis	Application of PRF * on implant stability	Implant stability Bone healing and formation	PRF can increase implant stability and may accelerate bone healing and promote new bone formation at the implant site.

* L-PRF = leucocyte platelet-rich fibrin; ISQ = implant stability quotient; PRF = platelet-rich fibrin.

**Table 5:** The characteristics and evaluation from included studies in periodontology.

Authors, Year Reference Journal Study Design	Application	Evaluation	Conclusions
Miron et al., 2020 [87] Clinical oral investigations Systematic review and meta-analysis	PRF for the treatment of gingival recession	Root coverage CAL Keratinized mucosa width (KMW) PD	PRF used with coronally advanced flap improved CAL and root coverage vs. CAF alone but did not improve KMW or PD.
Gusman et al., 2021 [88] Journal of clinical and experimental dentistry Systematic review and meta-analysis	PRF in palatal wounds following free gingival graft harvesting	Palatal wound Epthelialization Postoperative pain	PRF may decrease postoperative pain and induce earlier complete wound epithelialization.
Skurska et al., 2023 [89] Adv Med Sci Systematic review	PRF in class II furcation defects	Wound healing	The adjunctive use of platelet-rich fibrin in surgical treatment of furcation defects
Castro et al., 2017 [90] J Clin Periodontol. Systematic review and meta-analysis	L-PRF in periodontal surgery	PD CAL Bone fill Keratinized tissue width (KTW) Recession reduction Root coverage (%)	Significant PD reduction, CAL gain, and bone fill were found when comparing L-PRF to open flap debridement. For furcation defects, significant PD reduction, CAL gain, and bone fill were reported when comparing L-PRF to OFD. When L-PRF was compared to a connective tissue graft, similar outcomes were recorded for PD reduction, CAL gain, KTW, and recession reduction.
Meza-Mauricio et al., 2021 [91] Clin Oral Investig Systematic review	PRF after free gingival graft harvesting	Healing Control of pain Postoperative bleeding in palatal area after free gingival graft harvesting	The use of a PRF membrane for the protection of the palatal donor site following free gingival graft harvesting procedures improves wound healing and patients’ quality of life.
Panda et al., 2020 [92] Materials Systematic review and meta-analysis	L-PRF on coronally advanced flap (CAF) procedure in recession defects (Miller’s class I and II)	Gingival thickness (GT) Width of keratinized gingiva (WKG) Root coverage (%) CAL Recession depth (RD)	A significant improvement in GT, CAL, and RD was found when treated with CAF + L-PRF. L-PRF use in addition to CAF showed favorable results for the treatment of class I and II gingival recession defects.

CAL = clinical attachment loss; KMW = keratinized mucosa width; PD = probing depth; CAF = coronally advanced flap; PRF = platelet-rich fibrin; L-PRF = leucocyte platelet-rich fibrin; OFD = open flap debridement; PRP = platelet-rich plasma; GT = gingival thickness; WKG = width of keratinized gingiva; RD = recession depth.

**Table 6:** The characteristics and evaluation from included studies in ONJ.

Authors, Year Reference Journal Study Design	Application	Evaluation	Conclusions
Rusilas et al., 2020 [93] Stomatologija Systematic review	APC (PRF or PRP) in MRONJ	Mucosal integrity Absence of residual infection Presence of cutaneous fistulas Re-intervention necessary to healing Reduction in pain-VAS score evaluation	Faster wound closure (after 1 month) and decreased risk of infection in surgical site were observed in PRF group vs. control. Lower necessity of re-intervention was observed in PRF group as well as a lower VAS score was observed in PRF group after surgery.

APC = autologous platelet concentrates; PRF = platelet-rich fibrin; PRP = platelet-rich plasma.

**Table 7:** The characteristics and evaluation from included studies in regenerative.

Authors, Year Reference Journal Study Design	Application	Evaluation	Conclusions
Niu et al., 2018 [94] Implant dentistry Systematic review	PRP and L-PRP, PRF and L-PRF in alveolar ridge preservation	Alveolar width Alveolar height	Leukocyte- and platelet-rich fibrin might have a more positive effect on alveolar width and height preservation than PRP.
Miron et al., 2017 [95] Clinical oral investigations Systematic review	PRF in regenerative dentistry	PPD and CAL gain for intrabony defects Soft tissue generation Root coverage (%) for gingival recession CAL gain for furcation defects Dimensional change/density of hard tissue for bone regeneration	PRF leads to statistically superior periodontal repair (PPD and CAL gain) of intrabony defects when compared to OFD alone. The use of PRF led to a significant improvement in CAL when compared to controls (OFD alone), but the process can solely be defined as tissue “repair.” The use of PRF favors a slight gain in root coverage when compared to CAF alone, but further studies are needed to validate the potential advantages of the use of PRF for bone regeneration.
Miron et al., 2021 [96] Clinical oral investigations Systematic review and meta-analysis	PRF in periodontal intrabony defects	PD CAL gain Radiographic bone fill	The use of PRF in conjunction with open flap debridement statistically significantly reduced PD and improved CAL and RBF values.
Al-Maawi et al., 2021 [97] International journal of implant dentistry Systematic review	PRF in post-extraction sockets	Postoperative pain Wound healing Soft tissue regeneration Bone regeneration Bone loss	PRF significantly reduces postoperative pain (66.6% studies) and is most effective in the early healing period of 2–3 months after tooth extraction. Dimensional bone loss was lower in PRF group vs. spontaneous healing after 8–15 weeks. Socket fill was in 85% of cases and was higher in PRF group compared to spontaneous wound healing.
Moraschini et al., 2015 [98] International journal of oral and maxillofacial surgery Systematic review	APC in alveolar socket preservation	Healing Soft tissue epithelialization Postoperative pain and discomfort Hard tissue regeneration	The use of plasma concentrates seems to accelerate healing and soft tissue epithelialization in extraction sockets and reduce postoperative pain and discomfort. The use of PRF improved gain of keratinized gingiva after soft tissue surgery. Plasma concentrates reduce pain and inflammation and, therefore, provide more comfort postoperatively, but there is no evidence to date for their effect on bone regeneration.
Del Fabbro et al., 2014 [99] European journal of oral Systematic review and meta-analysis	APC in post-extraction socket healing	Hard and soft tissue healing Tissue regeneration Socket healing Postoperative complications Patient satisfaction Pain Swelling	There was a positive effect of platelet concentrates on soft tissue healing and the patient’s reported postoperative symptoms, like pain and swelling. Positive effects were also highlighted for bone formation, but the results need to be cautiously interpreted.
Del Fabbro et al., 2017 [100] Journal of oral and maxillofacial surgery Systematic review and meta-analysis	APC in post-extraction sockets	Soft tissue healing Swelling Trismus Incidence of alveolar osteitis Bone healing and remodeling	APCs should be used in post-extraction sites to improve clinical and radiographic outcomes, such as bone density and soft tissue healing, and postoperative symptoms (swelling, trismus), but their benefit is still not quantifiable in pain reduction.
Miron et al., 2017 [101] Tissue engineering Systematic review	PRF in soft tissue wound healing	Wound healing Soft tissue regeneration	PRF has positive effects on wound healing after regenerative therapy in various soft tissue defects.
Dragonas et al., 2019 [102] Int J. of Oral and Maxillofacial Surgery Systematic review	L-PRF in different intra-oral bone grafting procedures	Bone regeneration Soft tissue healing Postoperative complications	The use of L-PRF in extraction sockets was associated with a modest beneficial effect by decreasing alveolar ridge remodeling and postoperative pain when compared to natural healing. The use of L-PRF in maxillary sinus augmentation was not associated with more favorable outcomes.
Anitua et al., 2022 [103] Bioengineering Systematic review and meta-analysis	PRP (L-PRP and P-PRP) in post-extractive alveolar bone regeneration	New bone formation Bone density	A statistically significant difference was also observed in the P-PRP group for bone density outcome. The L-PRP treated sockets also showed higher bone density (SMD, 0.88; 95% CI, 0.31 to 1.45) in comparison to control sockets.
Pan et al., 2019 [104] J Am Dent Assoc. Systematic review and meta-analysis	PRF in alveolar ridge preservation	Postoperative pain Soft tissue healing Bone density Horizontal and vertical ridge dimension Alveolar osteitis Bone height Bone fill	PRF may play a positive role in reducing postoperative pain during the first week and ridge dimension changes after tooth extraction (6-month follow-up). PRF may be associated with smaller mesial bone height changes and more bone fill after tooth extraction, but further clinical studies are needed.
Lin et al., 2019 [105] Int J of oral and maxillofacial implants Systematic review and meta-analysis	PRF in ridge preservation	Bone healing Ridge height and width Osteoblastic activity Bone volume and density	PRF alone in ridge preservation does not provide significant additional benefits when compared to natural healing sockets with regard to bone volume, bone density, and osteoblastic activity.
Caponio et al., 2023 [106] Clin Oral Investig. Systematic review and meta-analysis	L-PRF and P-PRP in alveolar ridge preservation	Post-extraction socket healing Bone formation	In alveolar ridge preservation, the use of L-PRF and P-PRP is beneficial because any PC increases new bone formation compared to spontaneous healing.

APC = autologous platelet concentrates; PRF = platelet-rich fibrin; PRP = platelet-rich plasma; L-PRP = leucocyte platelet-rich plasma; L-PRF = leucocyte-rich fibrin; PPD = periodontal probing depth; CAL = clinical attachment loss; OFD = open flap debridement; CAF = coronally advanced flap; PD= probing depth; RBF = radiographic bone fill; PC = platelet concentrate.

**Table 8:** The characteristics and evaluation from included studies in endodontics.

Authors, Year Reference Journal Study Design	Application	Evaluation	Conclusions
Meschi et al., 2016 [107] Platelets Systematic review	APC in endodontic healing	Bone healing Soft tissue healing Postoperative quality of life Root development Pulp vitality	APCs in endodontic treatments seem to contribute to the healing of soft and hard tissues, improve the patients’ quality of life in the early postoperative period, aid further root development, and support maintenance or regaining of pulp vitality.

APC = autologous platelet concentrates.

**Table 9:** The characteristics and evaluation from included studies in orthodontics.

Authors, Year Reference Journal Study Design	Application	Evaluation	Conclusions
Farshidfar et al., 2022 [108] International orthodontics Systematic review and meta-analysis	APC (I-PRF) in orthodontic tooth movement	Orthodontic canine movement	I-PRF seems to be efficient in accelerating the orthodontic tooth movement of the canines, especially in the 2nd month.

APC = autologous platelet concentrates; I-PRF = injectable platelet-rich fibrin.

**Table 10:** The characteristics and evaluation from included studies in oral lesions.

Authors, Year Reference Journal Study Design	Application	Evaluation	Conclusions
Maddheshiya et al., 2023 [109] National journal of maxillofacial surgery Systematic review	PRP for oral lichen planus	Pain Lesion appearance	PRP can be considered a potential alternative therapy in treating non-responsive oral lichen planus, alleviating clinical signs and symptoms associated with oral lichen planus.
Gupta et al., 2024 [110] Cureus Systematic review and meta-analysis	i-PRF for oral lichen planus	Pain Surface area of lesions Patient satisfaction	i-PRF can be a potential treatment for oral lichen planus. The use of i-PRF resulted in pain reduction, lesion size improvement, and increased patient satisfaction.

PRP = platelet-rich plasma; I-PRF = injectable platelet-rich fibrin.

**Table 11:** Level of evidence of systematic reviews with meta-analysis included according to the AMSTAR 2 tool.

Gupta et al., 2024 [110]		✓		
Maddheshiya et al., 2023 [109]			✓	
Farshidfar et al., 2022 [108]			✓	
Meschi et al., 2016 [107]		✓		
Caponio et al., 2023 [106]				✓
Lin et al., 2019 [105]		✓		
Pan et al., 2019 [104]	✓			
Anitua et al., 2022 [103]		✓		
Dragonas et al., 2019 [102]			✓	
Miron et al., 2017 [101]		✓		
Del Fabbro et al., 2017 [100]			✓	
Del Fabbro et al., 2014 [99]	✓			
Moraschini et al., 2015 [98]		✓		
Al-Maawi et al., 2021 [97]		✓		
Miron et al., 2021 [96]			✓	
Miron et al., 2017 [95]				✓
Niu et al., 2018 [94]			✓	
Rusilas et al., 2020 [93]		✓		
Panda et al., 2020 [92]			✓	
Meza-Mauricio et al., 2021 [91]				✓
Castro et al., 2017 [90]			✓	
Skurska et al., 2023 [89]				✓
Gusman et al., 2021 [88]			✓	
Miron et al., 2020 [87]				✓
Guan et al., 2023 [86]		✓		
Lyris et al., 2021 [85]			✓	
Riberio et al. 2024 [84]		✓		
Zhu et al., 2021 [83]			✓	
Campana et al., 2023 [82]				✓
Canellas et al., 2017 [81]		✓		
Xiang et al., 2019 [80]			✓	
Ramos et al., 2022 [79]	✓			
Bao et al., 2021 [78]			✓	
Vitenson et al., 2022 [77]		✓		
He et al., 2017 [76]			✓	
Salgado-Peralvo et al., 2022 [75]			✓	
Al-Hamed et al., 2017 [74]			✓	
Franchini et al., 2019 [73]		✓		
Bao et al., 2021 [72]			✓	
Filho et al., 2021 [71]		✓		
	No or one non-critical weakness: the systematic review provides an accurate and comprehensive summary of the results of the available studies that address the question of interest.	Weakness: the systematic review has more than one weakness but no critical flaws. It may provide an accurate summary of the results of the available studies that were included in the review.	Without non-critical weaknesses: the review has a critical flaw and may not provide an accurate and comprehensive summary of the available studies that address the question of interest.	More than one critical flaw with or without non-critical weaknesses: the review has more than one critical flaw and should not be relied on to provide an accurate and comprehensive summary of the available studies.
